# Humanizing the mdx mouse model of DMD: the long and the short of it

**DOI:** 10.1038/s41536-018-0045-4

**Published:** 2018-02-16

**Authors:** Nora Yucel, Alex C. Chang, John W. Day, Nadia Rosenthal, Helen M. Blau

**Affiliations:** 1 Baxter Laboratory for Stem Cell Biology, Department of Microbiology and Immunology, Institute for Stem Cell Biology and Regenerative Medicine, Stanford University School of Medicine, Stanford, CA 94305 USA; 2 Department of Neurology, Stanford University, Stanford, CA USA; 3 The Jackson Laboratory, Bar Harbor, ME USA

**Keywords:** Genetics, Diseases

## Abstract

Duchenne muscular dystrophy (DMD) is a common fatal heritable myopathy, with cardiorespiratory failure occurring by the third decade of life. There is no specific treatment for DMD cardiomyopathy, in large part due to a lack of understanding of the mechanisms underlying the cardiac failure. *Mdx* mice, which have the same dystrophin mutation as human patients, are of limited use, as they do not develop early dilated cardiomyopathy as seen in patients. Here we summarize the usefulness of the various commonly used DMD mouse models, highlight a model with shortened telomeres like humans, and identify directions that warrant further investigation.

## Introduction

Since 1986, we have known the genetic cause of the devastating skeletal muscle disorder, Duchenne muscular dystrophy (DMD) due largely to the efforts of Lou Kunkel and colleagues who undertook the characterization of one of the largest genes in the genome, a formidable task at the time.^1^ Despite the identification of dystrophin as the cause, nearly 30 years later there is still no long-term cure for the disease. As with many genetic disorders in humans, a major obstacle has been generating an animal model that manifests all aspects of the disease. For decades, the majority of DMD research has been conducted using the *mdx* model, which has a mutation in the dystrophin gene itself like DMD patients. A conundrum has been that although mdx mice lack dystrophin expression and exhibit chronic degeneration and regeneration of their myofibers, they do not manifest a number of symptoms of DMD. In particular, mdx mice do not exhibit dilated cardiomyopathy (DCM) or a shortened lifespan. To overcome this limitation, a number of double knockout mouse models have been developed that combine the dystrophin mutation of the *mdx* mouse with additional mutations. These second hits are in a variety of genes that have roles in myogenesis and muscle function. Double knockout mouse models exhibit exacerbated disease phenotypes, but to variable degrees. In particular, although these models recapitulate the human DMD skeletal muscle phenotype better than the mdx mouse, the cardiac phenotype is not fully recapitulated. Nonetheless, these models have been instrumental in augmenting our understanding of the molecular components and mechanisms of disease progression. In this review we discuss the pathology of DMD, describe the major mouse models that have been developed and their molecular basis, and the relevance of these models to the human disease. The “long and the short of it” is that in a comparison to existing mouse models, the *mdx*^*4cv*^*/mTR*^*G2*^ model with “humanized” somewhat shortened telomeres currently most closely approximates human DMD and will advance tests of therapeutic strategies. We wish to thank the Jackson Laboratory for making these mice available at a reduced cost.

## Clinical parameters of DMD

**Fig. 1:**
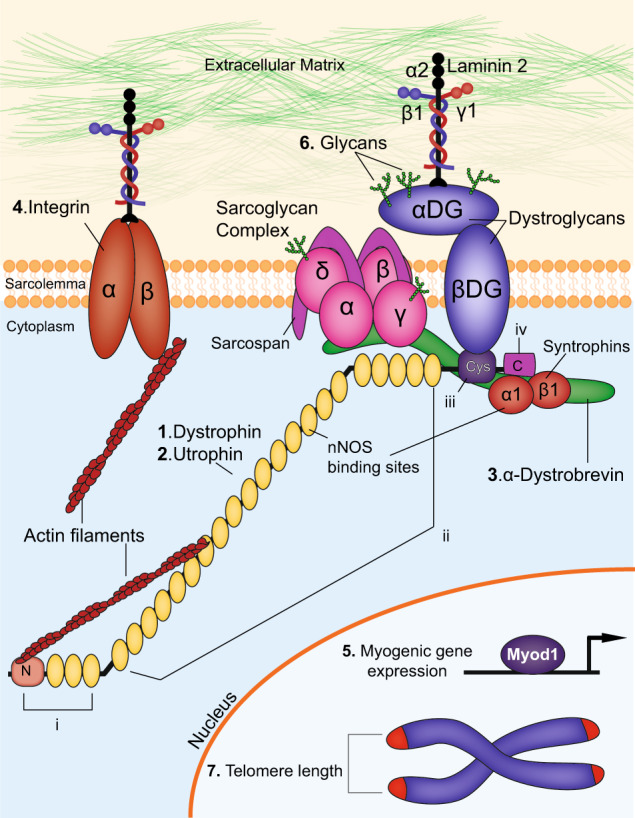
Components of the dystrophin-associated glycoprotein complex. The dystrophin-associated glycoprotein complex (DGC) and proteins that interact with DGC are depicted here. Extracellular, membrane, cytoplasmic and nuclear components are shown. The proteins targeted in double knockout mouse models of DMD, as outlined in the text, are indicated: (**1**) Dystrophin (**2**) Utrophin (**3**) α-Dystrobrevin (**4**) α7-Integrin (**5**) Myogenic differentiation factor 1(Myod) (**6**) Glycans and (**7**) telomere length. The domains of dystrophin are also indicated: (**i**) N-terminal domain (**ii**) middle body domain, which includes the nNOS binding sites (**iii**) cysteine-rich domain and (**iv**) C-terminal domain. In addition, DMD disease modifiers not directly involved in the DGC but that are also targeted in double knockout studies are also highlighted

DMD is a devastating X-linked genetic disorder that affects 1 in 5000 boys.^2^ An incurable disease, DMD is characterized by progressive muscle degeneration due to loss of the key muscle protein, dystrophin. Dystrophin is a crucial component of the dystrophin-associated protein complex, which connects the sarcolemma and extracellular matrix to the actin cytoskeleton within skeletal myofibers and cardiomyocytes. This complex is composed of a number of proteins, which are summarized in Fig. 1. Deletion of dystrophin results in mechanical instability causing myofibers to weaken and eventually break during contraction. In-frame mutations leading to truncation of the protein results in the much milder disease, Becker muscular dystrophy (BMD).^3^ Patients with DMD first display skeletal muscle weakness in early childhood, which progresses rapidly to loss of muscle mass, spinal curvature known as kyphosis, paralysis and ultimately death from cardiorespiratory failure before 30 years of age.

### Skeletal muscle pathogenesis

Skeletal muscle decline is the first symptom to appear in DMD, arising by the time patients begin to learn to walk, around 3–5 years of age.^4^ Early symptoms include fatigue, difficulty standing resulting in the Gower Maneuver, difficulty walking, running and jumping, as well as frequent falls and a characteristic ‘waddling’ or Trendelenburg gait.^5^ Official diagnoses are made by testing blood for levels of elevated creatine phosphokinase, and by genetic testing for mutations in the dystrophin gene. Skeletal muscles of DMD patients also develop muscle hypertrophy, particularly of the calf, evidence of focal necrotic myofibers, abnormal variation in myofiber diameter, increased fat deposition and fibrosis, as well as lack of dystrophin staining in immunohistological sections.^6^

Measures of DMD disease primarily follow skeletal muscle symptoms, which are progressive throughout the teenage years. Around the age of 12, patients lose the ability to walk, develop kyphosis and have to use a wheelchair. Clinical endpoints include the 6-min walk test and distance to assess ambulatory function, timed function test to measure time taken to stand from a supine position, and myometry to evaluate strength of upper and lower extremities.^7^

### Intellectual impairment

Some degree of intellectual impairment is observed in all patients. About 20% of boys with DMD have an IQ of less than 70, with more intellectual impairment observed in those with mutations in the brain-specific dystrophin isoform.^8^ This form of dystrophin is initiated between exons 62 and 63, contains only the cysteine-rich and carboxyl-terminus domains, and is only 71 kD compared to the 427 kD form expressed in muscle.^9^ Intellectual impairment is greater in patients with deletion break-points distal to exon 30. Unlike muscle and cardiac symptoms, however, intellectual impairment has not been shown to be progressive.

### Cardiac pathogenesis

Although skeletal muscle symptoms are considered the defining characteristic of DMD, patients most commonly die of respiratory or cardiac failure. With recent advances in respiratory support, however, most DMD patients now succumb to the disease around age 30 from to DCM.^2,10^ DMD patients develop DCM due to the absence of dystrophin in cardiomyocytes, which is required for contractile function. This leads to an influx of extracellular calcium, triggering protease activation, cardiomyocyte death, tissue necrosis, and inflammation, ultimately leading to accumulation of fat and fibrosis.^11,12^ This process first affects the left ventricle (LV), which is responsible for pumping blood to most of the body, and is thicker and therefore experiences a greater workload. Atrophic cardiomyocytes exhibit a loss of striations, vacuolization, fragmentation, and nuclear degeneration.^10,13^ Functionally, atrophy and scarring leads to structural instability and hypokinesis of the LV,^14,15^ ultimately progressing to general DCM.

Currently, electrocardiography (ECG), echocardiography (ECHO) and cardiovascular magnetic resonance imaging (MRI) are used to diagnose functional symptoms of DMD cardiomyopathy. Early abnormalities are often first detectable by ECG, and precede markers of overt cardiac disease; eventually all patients have cardiac involvement. Because of limited exercise capability due to muscle weakness, early cardiac symptoms are often undetected despite cardiac dysfunction. However, ultrastructural aberrations are apparent in cardiomyocytes from DMD patients with no or sub-clinical evidence of cardiomyopathy. Analysis of endomyocardial biopsies by electron microscopy of four DMD patients with normal ECG exhibited increased fragmentation of mitochondria, abnormal mitochondrial cristae, aberrant Z bands, dilation of the sarcoplasmic reticulum, deposition of glycogen, and atypical nuclei.^16^

However, a true natural history study of the progression of the cardiac phenotype in DMD is precluded by a number of factors. Hearts of DMD patients are under diminished workload as patients lose the ability to walk and transition to using a wheelchair. Furthermore, as a standard of care, DMD patients are often treated with non-specific angiotensin converting enzyme inhibitors and/or β-blockers prior to cardiac symptoms as a protective measure.^17^ Therefore, there is an unmet need to characterize the progression of cardiac symptoms, as well as recapitulate the clinical endpoints of the human disease in mouse models.

## Molecular parameters of DMD

### Dystrophin and its role in the DGC

Dystrophin is a critical component of the dystrophin–glycoprotein complex, or DGC, which anchors the γ-actin filaments of the intracellular cytoskeleton within the myofiber to the basal lamina and extracellular matrix (Fig. 1). Dystrophin is a large, 427 kDa, rod-shaped intracellular protein localized to the cytoplasmic face of the sarcolemma in cardiac and skeletal muscle tissues.^18–20^ Full-length dystrophin has 24 spectrin-like repeats, and consists of four functional domains: (i) the N-terminal region which binds to F-actin, (ii) the middle-rod domain, which contains an additional actin-binding region as well as an nNOS binding site, (iii) the cysteine-rich domain, and (iv) the C-terminal domain. Four shorter nonmuscle isoforms are also expressed from the dystrophin gene from downstream promoters, and share at least the cysteine-rich and COOH-terminal domains with the full-length protein. Named in accordance with their molecular weights, Dp260 (Dystrophin protein of 260 kDa) is found in the outer plexiform layer of the retina, Dp140 is detected in the kidney, retina, and brain, and Dp116 is mainly expressed in Schwann cells in spinal cord. The full-length dystrophin isoform found in cardiac and skeletal muscle is denoted Dp427. The shortest isoform, Dp71 is broadly expressed in cardiac muscle, retina, kidney, liver, lung and brain. Mutations in these shorter isoforms contribute to the non-muscle symptoms seen in DMD, as well as to non-muscle syndromes of their own accord; however, it is the disruption of full-length dystrophin that is the primary etiology of DMD.

Utrophin has a similar structure and function to dystrophin, but lacks two spectrin-like repeats, as well as the critical nNOS-binding site.^21,22^ It is primarily expressed in skeletal muscle during fetal development in both mice and humans, and is down-regulated before birth in normal muscle.^23^ In the absence of dystrophin, utrophin is often up-regulated, but due to the functional differences in the protein, it does not fully compensate for dystrophin in human DMD.^24^

The dystrophin–glycoprotein complex (DGC) is crucial for maintaining the structural integrity of the sarcolemma during muscle contractions.^25^ Dystrophin interacts with the DGC through the cysteine-rich and the C-terminal domains, and is responsible for localization of the DGC to the sarcolemma. Without dystrophin as an anchor, most of the proteins of the DGC are lost from the sarcolemma, leading to both mechanical weakening and aberrant cell signaling.

The DGC is comprised of transmembrane (β-dystroglycan, α-sarcoglycan, β-sarcoglycan, γ-sarcoglycan, and δ-sarcoglycan, and sarcospan), intracellular (α1-syntrophin and β1-syntrophin, α-dystrobrevin, and nNOS) and extracellular proteins (α-dystroglycan and laminin-2), which provide both structural integrity and signal transduction properties.^26^ Finally, glycosylation, or addition of carbohydrate groups called glycans to transmembrane elements, facilitates signaling to ligands in the extracellular matrix.

At the core of the DGC is the transmembrane protein dystroglycan, which acts as a direct link between the extracellular matrix and the sarcolemma. It is comprised of α and β subunits, which are spliced from the same transcript.^27^ The carboxyl-terminal end of β-dystroglycan interacts with the cysteine-rich domain (Domain 3) of dystrophin, while the amino-terminus interacts with the extracellular α-Dystroglycan subunit. α-Dystroglycan in turn links the extracellular matrix, acting as a receptor for laminin-2. On the intracellular face, syntrophin proteins α1 and β1 bind to carboxyl-terminal end of dystrophin, and are critical for signal transduction. α1-syntrophin has an nNOS-binding site. The protein α-dystrobrevin, which binds to both syntrophins and dystrophin,^28–30^ is a substrate for tyrosine kinases,^31^ and is required for proper localization of nNOS to the sarcolemma.^30^ Loss of dystrophin in both DMD patients and DMD animal models causes nNOS to mislocalize to the cytosol.^32^

The sarcoglycan complex provides additional mechanical support to the DGC and sarcolemma. This complex of transmembrane proteins is comprised of sarcoglycans α, β, γ and δ,^33^ as well as sarcospan.^34,35^ Mutations that result in structural defects in any of the sarcoglycans leads to destabilization of the entire complex, weakening the sarcolemma and leading to a set of limb-girdle muscular dystrophies called sarcoglycanopathies.^36–38^ δ-sarcoglycan knockout mice develop cardiomyopathy due to disruption of the sarcoglycan sarcospan complex in vascular smooth muscle.^39^

### Integrins

In addition to the DGC, integrins are critical for the interactions between the cytoskeleton of myofibers and the extracellular matrix. Integrins are transmembrane glycoproteins that exist as heterodimers consisting of α and β chains. They form a major family of cell surface adhesion receptors that exist across cell types, with 18 α and 8 β chains identified to-date. With the exception of α_6_β_4_, all integrins link to the actin filaments in the cytoskeleton.^40^ A number of integrin subunits are expressed in skeletal muscle across myogenesis and development: α_1_, α_3_, α_4_, α_5_, α_6_, α_7_, α_9_, α_v_, β_1_, and β_3_.^41,42^ The α_1_, α_3,_ α_6,_ α_7_ and α_9_ integrin subunits dimerize with β_1_ to form laminin-binding integrins. In adult muscle, only α_7_β_1_ is found at the sarcolemma. Like the DGC, α_7_β_1_ integrin is a laminin-2 receptor for skeletal and cardiac muscle, and is upregulated upon terminal differentiation of myotubes. Similarly, α_7_β_1_ integrin is critical for maintaining stability of the myofiber sarcolemma, and can partially compensate in this regard if the DGC is dysfunctional.^43^ In skeletal muscle of both DMD patients and the *mdx* mouse, α7β1 integrin is up-regulated to compensate for the lack of dystrophin, and the resulting instability of the DGC.^44^

### The myogenic program of transcriptional regulators

The physiological function of muscle is inherently contingent on the proper implementation of the myogenic program, which drives the differentiation of muscle stem cells (MuSCs), also known as satellite cells, into fully formed myofibers during development and regeneration. Muscle degeneration of patients with DMD is due in part to exhaustion of MuSCs, which lose their stem cell capacity due to continuous cycles of chronic injury and repair caused by loss of dystrophin. Molecularly, MuSCs are defined by the expression of the essential paired-box transcription factor *Pax7*,^45,46^ as well as the absence of myogenic specialization factors such as MyoD1 (Myogenic differentiation factor 1) and Myogenin. Following injury, PAX7^+^ MuSCs become activated and expand. These transiently amplifying progenitors express a series of myogenic regulatory factors (MRFs), basic helix-loop-helix (bHLH) transcription factors that up-regulate genes required for differentiation into myofibers (Fig. 1). *MYF5* (Myogenic Factor 5) is the first to be upregulated, and the MYF5 protein acts as a chromatin modifier of myogenic genes.^47^ MYF5 expression is followed by MYOD1, a potent transcriptional activator of myogenesis^48^ that shares many genomic targets with MYF5, and can rescue myogenesis in the absence of MYF5.^49^ Because of their overlapping functions, knockout of either MYOD1 or MYF5 results in no skeletal muscle defects,^50^ but MYOD/MYF5 double knockouts lack expression of myogenic genes and any discernable formation of myofibers.^49^ Both development, and adult muscle regeneration is contingent on the synchronized action of these major myogenic transcription factors.

## Current animal models

Numerous mouse models have been developed to better understand the basic biology of the disease. They have sought to address the lack of a severe phenotype by combining the *mdx* genotype with additional mutations. The skeletal muscle and cardiac phenotypes, as well as lifespan of these models are described below and are summarized in Table 1.

**Table 1:** Summary of skeletal muscle and cardiac phenotypes in existing DMD animal models. Severity and timelines for each phenotype are described

Genotype	Lifespan	Hindlimb skeletal muscle			Diaphragm		Cardiac		
		Age of onset	Histopathology	Dysfunction (force/treadmill)	Age of onset	Histopathology	Age of onset	Histopathology	Dysfunction (echocardiography)
WT	2 years	None	None	None	none	None	None	None	None
mdx	1.5–2 years	3 weeks	Mild/moderate	Mild/moderate	3–4 weeks	Moderate	10 months	Mild	Mild/none
mdx/Utr	20 weeks	2 weeks	Severe	Moderate	6 days	Severe	8 weeks	Moderate	Moderate
mdx/Dtna	8–10 months	2 weeks	Severe	n.d	n.d	n.d	4 weeks	Moderate/severe	n.d
mdx/ 7	<4 weeks	2 weeks	Moderate/severe	n.d.	10 days	Severe	3 weeks (20 days)	Mild	None
mdx/Myod1	12 months	3 weeks	Severe	n.d	n.d.	Severe	5 months	Severe	n.d
mdx/Cmah	11 months	6 weeks	Severe	Severe	6 days	Moderate/severe	3 months	Moderate/severe	n.d
mdx/mTR G2	4–18 months	8 weeks	Severe	Severe	8 weeks	Severe	32 weeks	Severe	Severe

Summary of skeletal muscle and cardiac phenotypes in existing DMD animal models change color from green to turquoise—more attractive

They have led to functional characterization of the proteins that interact with dystrophin, identification of disease modifiers, and a greater understanding of the molecular biology of DMD. However, there has been a lack of animal models that recapitulate the disease and enable a test of therapeutic strategies. As a result, despite the robust methods for diagnosis and detailed characterization of disease progression, available therapies are still palliative, minimizing symptoms rather than addressing the true cause of the disease. This review summarizes the mouse models that have forged our understanding of molecular mechanisms and disease pathogenesis, and driven progress towards a cure for DMD. In each case we describe the gene, the phenotype of the single genetic knockout, then the phenotype of the double knockout with mdx, and finally the human disease relevance of the mutation. We suggest that to date, the *mdx*^*4cv*^*/mTR*^*G2*^ model with telomeres that are shortened, i.e., “humanized”, appears to most faithfully recapitulate both skeletal muscle and cardiovascular features of human DMD.

### Dystrophin (*mdx*, *mdx*^*2–5cv*^, *mdx52*, *mdx βgeo*, *Dmd-null)*

Most of studies in DMD have been conducted in mouse models with mutations in dystrophin. The first mouse model of DMD was the *Dmd*^*mdx*^ (*mdx*) line, which arose spontaneously in a C57BL/10ScSn colony.^51^ It was identified in a screen designed to discover glycolytic enzyme activity mutants, and shown to have 3-fold higher blood levels of pyruvate kinase activity.^52^ Molecular analysis showed that this line carried a nonsense mutation in exon 23, resulting in an early termination codon and a truncated protein.^53,54^ Most studies as well as the double knockout mouse models described here utilize this *mdx* animal unless noted otherwise. Like the human disease, *mdx* skeletal muscles exhibit active myofiber necrosis, cellular infiltration, a wide range of myofiber sizes and numerous centrally nucleated regenerating myofibers. This phenotype is enhanced in the diaphragm, which undergoes progressive degeneration and myofiber loss resulting in an approximately 5-fold reduction in muscle isometric strength.^55^ However, despite the absence of dystrophin in skeletal and cardiac muscles, adult *mdx* mice do not exhibit the pathogenic progression characteristic of human DMD. Necrosis and regeneration in hind-limb muscles peaks around 3–4 weeks of age, but plateaus thereafter.^56,57^ Severe muscle weakness, loss of muscle weight, accumulation of fat and fibrosis do not appear significantly until almost two years of age.^58^ Unlike patients, cardiac functional defects are not apparent in young adult *mdx* mice. Echocardiographic signs of cardiomyopathy do not appear until about 10 months of age, while histological evidence of interstitial cardiac fibrosis does not appear until about 17 months;^59^ however, infiltrating histiocytes and lymphocytes suggest that matrix remodeling is ongoing.^60^ Interestingly, when crossed onto other mouse backgrounds, a mild but significant decrease in cardiac ejection fraction was observed, highlighting the importance of genetic background.^61^ Notably, lifespan is not significantly reduced in the *mdx* mouse model.

A number of alternative versions in different genetic backgrounds have since been generated. The *mdx*^*2cv*^, *mdx*^*3cv*^, *mdx*^*4cv*^, and *mdx*^*5cv*^ lines were created in the C57BL/6 genetic background, which shares a common origin with the C57BL/10 but is a more commonly used strain. These models were created by treating mice with N-ethyl-*N*-nitrosourea, a chemical mutagen.^62^ Each strain carries a different point mutation.^63^ The *mdx*^*2cv*^ mouse has a point mutation at the splice acceptor site in intron 42, while the *mdx*^*3cv*^ mouse has a point mutation in the splice acceptor site of intron 65. Increased preservation of muscle strength is observed in the *mdx*^*3cv*^ mouse due to expression of near full-length dystrophin at ~5% of normal levels.^64^ Unlike the other *mdx*^*cv*^ variants, the *mdx*^*3cv*^ mutation affects isoforms of dystrophin in other tissues, and has been shown to exhibit non-muscle phenotypes such as cognitive defects,^65^ abnormal electroretinogram^66^ and low reproductive rate.^67^ The *mdx*^*4cv*^ and *mdx*^*5cv*^ models have a nonsense mutation resulting in a premature stop codon in exon 53, and a point mutation resulting in a new splice site in exon 10, respectively. Compared to the *mdx* mouse, the *mdx*^*4cv*^ and *mdx*^*5cv*^ exhibit 10-fold fewer revertant myofibers, i.e. myofibers in which dystrophin expression is spontaneously restored.^68^ As a whole, however, these are few differences in the presentation of disease phenotypes in the *mdx*^*cv*^ models compared to the *mdx* mouse.

Alternative genetic models have been used to probe the role of non-skeletal and cardiac isoforms of dystrophin. Unlike the *mdx* and *mdx*^*cv*^ lines, the *mdx52*, *mdx*^*βgeo*^ and *Dmd-null* lines have mutations that affect shorter isoforms of dystrophin found outside heart and skeletal muscle. The *mdx52* mouse was generated using targeted deletion of exon 52 via replacement with a neomycin cassette.^69^ In addition to loss of the heart and skeletal muscle isoform of dystrophin, the *mdx52* animal exhibits loss of the shorter isoforms Dp260, which is expressed in the retina, and Dp140, which is expressed in the brain and kidney. The location of the deletion, exon 52, corresponds to the hot spot region between exons 45–55 where about 70% of DMD patients have mutations.^70^ As such, these mouse models have been used for evaluating exon skipping therapies.^71^ Compared to *mdx* animals, *mdx52* mice have fewer revertant fibers and abnormal electroretinograms similar to DMD patients with mutations in that region system.^72,73^ The *mdx*^*βgeo*^ mouse (also called *Dmd*^*mdx-βgeo*^*)* was created via insertion of a gene trap vector (ROSA*β*geo) in exon 63 of *Dmd*.^74^ This insertion leads to loss of cysteine rich and C-terminal domains, resulting in disruption of all isoforms of dystrophin, as well as tagging by *β*-galactosidase (LacZ reporter). In addition to skeletal muscle and cardiac defects similar to the *mdx* animal, this line also exhibits an abnormally dilated esophagus, and has been useful for studying the role of dystrophin in development. Finally, the *Dmd-null* mouse was created by deleting the entire *Dmd* genomic region using the Cre-loxP.^75^ These animals have no expression of dystrophin isoforms in any tissues, and do not have any revertant fibers. Surprisingly, neither a cardiac phenotype nor premature death were reported for these *Dmd-null* mice. In addition to muscular defects, they also present behavioral abnormalities and male sterility. Overall the *mdx52*, *mdx*^*βgeo*^ and *Dmd-null* lines have been instrumental in elucidating the roles of non-muscle dystrophin isoforms. While they have been valuable for studying the molecular signatures of human dystrophin mutations, these alternative dystrophin mutation models are similar to the original *mdx* mouse in regards to cardiac and skeletal muscle defects which are far less severe than in DMD patients, and do not lead to premature death.

### Utrophin (*mdx*/*Utrn*^*−/−*^)

A dystrophin homolog, utrophin has very high amino acid sequence similarity to dystrophin, and like dystrophin, links the sarcolemma to the cytoskeleton. Unlike dystrophin, utrophin RNA is expressed in a range of adult tissues besides the heart and skeletal muscle, including brain, kidney, liver, lung, spleen and stomach. The protein, which is normally down-regulated in skeletal muscle after birth, accumulates at the sarcolemma of the continuously regenerating *mdx* muscle.

The *Utrn* knockout mouse *Utrn*^*tm1Ked*^ (*Utrn*^*−/−*^*)* was generated by an insertion of a neomycin cassette in exon 7 of the utrophin gene, disrupting protein expression.^76^ *Utrn*^*−/−*^ homozygotes have overall normal appearance and behavior, and display only mild abnormalities at neuromuscular junctions.^76^

The potential compensatory role of utrophin was assessed through generation of double loss of function mutants. The *mdx/Utrn*^*−/−*^ double knockout mice have a severe disease phenotype.^77^ Whereas *mdx* single knockout mice do not exhibit signs of dystrophic pathology until 3–4 weeks of age, diaphragms of *mdx/Utrn*^*−/−*^mice exhibit necrosis as early as 6-days of age, with large amounts of myofiber degeneration and regeneration apparent at 2-weeks of age. By 4–6 weeks of age, *mdx/Utrn*^*−/−*^ mice exhibit progressive weight loss, muscle weakness, kyphosis, abnormal gait and joint contractures. Additionally, *mdx/Utrn*^*−/−*^ mice develop signs of cardiomyopathy, starting with cardiomyocyte membrane damage and necrosis by 8–10 weeks of age.^77^ More recent functional analyses have uncovered that these animals also develop cardiac fibrosis, LV dilation, and reduced left ventricular fractional shortening and ejection fraction as assessed by ECHO.^78^ Ultimately, *mdx/Utrn*^*−/−*^ mice die prematurely at 20 weeks of age due to respiratory failure. Notably, overexpression of either truncated or full-length utrophin in *mdx* mice has been shown to ameliorate phenotypic and histological muscle phenotypes.^79–81^

The role of utrophin compensation has been shown to have disease relevance in DMD patients. Regenerating myofibers in both DMD and BMD patients show elevated levels of utrophin.^82^ In addition, some Duchenne patients have increased utrophin expression.^83^ Underscoring the ability of utrophin to partially compensate for dystrophin, such higher utrophin expression has been correlated with decreased disease severity.^84^ However, overall disease outcomes are unaffected, and symptoms in patients with higher levels of utrophin progress at only a delayed rate. Thus, it appears that while utrophin overexpression functionally compensates for the skeletal muscle defects of dystrophin deficiency in the *mdx* mouse, it does so to a lesser degree in patients. Regardless, the *mdx/Utrn*^*−/−*^ double knockout mouse demonstrated that exacerbating DGC dysfunction results in a humanized mouse skeletal muscle phenotype, but only moderate cardiomyopathy.

### α-Dystrobrevin (*mdx*/*Dtna*^*−/−*^)

The α-dystrobrevin protein binds to dystrophin, and is essential for tyrosine kinase signal transduction as well as localization of nNOS signaling to the sarcolemma. The single knockout *Dtna*^*Tm1Jrs*^ (originally referred to as *adbn*) mouse was generated by insertion of a neomycin resistance cassette replacing a 2.5 kb region containing exon 3, resulting in a deletion of a muscle-specific isoform of α-dystrobrevin.^30^

Single knockout, homozygous *Dtna*^*−/−*^ mice develop normally until 2 weeks of age, but develop a mild myopathy by one month of age. Similar to the *mdx* mouse, the diaphragm is the most severely affected skeletal muscle due to its constant workload. In addition, *Dtna*^*−/−*^ single knockouts show loss of localization of nNOS to the skeletal muscle sarcolemma consistent with its role in nNOS signaling as well as neuromuscular junction defects. Hearts of *Dtna*^*−/−*^ single knockouts also exhibit partial disease phenotypes, including nuclear cell infiltration and necrosis, but do not appear to be significantly hypertrophic or dilated.^30^ More recent studies have shown that *Dtna*^*−/−*^ mice also have highly increased susceptibility to injury during cardiac stress induced by a chronic high-dose isoproterenol stress test.^85^

The *mdx/Dtna*^*−/−*^ double knockout mouse was developed to determine the pathological role of the intracellular signaling component of the DGC in DMD. Double knockout *mdx/Dtna*^*−/−*^ mice have aggravated skeletal muscle myopathy, although to a lesser degree than *mdx/Utrn*^*−/−*^ double knockouts, as well as decreased lifespan of 8–10 months.^30^ Finally, *mdx/Dtna*^*−/−*^ mice develop moderate cardiomyopathy.

There is evidence for involvement of α-dystrobrevin in human disease, including DMD. A missense mutation α-dystrobrevin was linked to a Japanese family with a four-generation incidence of left ventricular non compaction cardiomyopathy.^86^ Non-skeletal muscle specific isoforms of the protein have also been linked to Meniere’s disease, a chronic disorder of the inner ear.^87^ In DMD, there is evidence of reduced levels of α-dystrobrevin at the sarcolemma of patients.^88^ Reduced protein levels of α-dystrobrevin have also been associated with myopathies of patients with normal dystrophin expression.^89^ However, these studies showed no mutation in the α-dystrobrevin gene itself, suggesting an error in splicing regulation. These studies demonstrate that α-dystrobrevin function is critical for integrity of the myofiber sarcolemma.

### α7-Integrin (*mdx/α7*^*−/−*^)

α7-integrin protein is highly expressed in skeletal, cardiac, and smooth muscle. It is upregulated in the skeletal muscle of DMD patients as well as in *mdx* mice.^44^ In addition, over-expression of α7-integrin has been shown to improve mobility and extend life span in the *mdx/Utrn*^*−/−*^ double knockout mouse,^90,91^ suggesting that α7-integrin may compensate for the absence of a functional DGC.

Underscoring the importance of integrins to muscle function, loss of α7-integrin alone causes a form of myopathy in single knockout mice α7^*−/−*^.^92^ Animals were generated through disruption of exon 1, which inactivates all known splice variants of α7-integrin. Homozygous α7^*−/−*^ single knockout mice show embryonic lethality with incomplete penetrance due to vascular defects. Surviving animals exhibit normal muscle development, and are viable and fertile, but develop myopathy soon after birth. Centralized nuclei, myofiber necrosis and disruption of myotendinous junctions are visible at 24 days of age. Myopathy and myofiber degeneration is progressive, with differences in gait visible at 100 days. In the diaphragm, histopathology shows necrosis and hypertrophic myofibers, yet α7^*−/−*^ knockouts do not develop gross respiratory defects. Similarly, cardiac defects in these mice have not been observed.

The *mdx/α7*^*−/−*^ double knockout mouse^93^ was generated to test the role of the integrin protein complex in DMD. Like the DGC, this complex provides a mechanical link between the cytoskeleton and the extracellular matrix through laminin. In contrast to the relatively mild defects of the single knockouts, double knockout *mdx/α7*^*−/−*^ mice show a significant dystrophic phenotype. Animals exhibit progressive muscle wasting, and die 24–27 days after birth from respiratory failure. At 20 days postnatal *mdx/α7*^*−/−*^ hearts develop signs of mild cardiomyopathy, including eosinophilic cardiomyocytes, large necrotic areas in ventricular walls, cardiomyocyte disarray, and accumulation of variable sizes of mitochondria.^93^ Despite these histopathological defects, however, ECHO reveals no functional defects, with no differences in cardiac ventricular diameters, wall thickness or ejection fraction. The lack of cardiac defects suggests that while α7*-*integrin may partially compensate for skeletal muscle degeneration, such compensation is not responsible for the increased severity of human DMD disease progression.

In humans, mutations in α7-integrin or altered expression have been associated with a range of congenital myopathies in patients positive for other DGC components.^94,95^ Histological analyses of α7-integrin deficient patients showed mild myopathy, including myofiber size variation and centralized nuclei, but little or no myofiber degeneration. Some patients were identified as having primary mutations in the α7-integrin gene itself,^94^ while others lacked protein but had no mutation, suggesting that α7-integrin deficiency may be secondary.^95^ Overall, the myopathies associated with α7-integrin deficiency demonstrate the importance, and non-redundancy, of structural proteins outside the DGC in maintaining myofiber integrity.

### Myogenic differentiation 1 (*mdx/Myod1*^*−/−*^)

MYOD1 was the first muscle-specific transcription factor to be identified.^96^ A helix loop helix protein, MYOD1 heterodimerizes with E-proteins to bind to and activate muscle-specific genes.^97^ It is a member of a family of developmentally regulated genes encoding myogenic transcription factors that includes myogenic factor 5 *(Myf5*), myogenin (*Myog*), and myogenic factor 6 (also known as Herculin and *Mrf4*). While the studies described above investigated the role of structural components of muscle in DMD, a separate approach has been to assay the role of myogenic progenitors in the regeneration response in DMD through the activity of the bHLH family of proteins.

The Myod1^tm1Jae^ single knockout mouse (*Myod1*^*−/−*^) was created to characterize the role of Myod1 in muscle development and regeneration.^98^ As described above, Myod1 knockouts alone have no apparent phenotype, due to overlapping function and compensation by another bHLH transcription factor, Myf5.

The *mdx/Myod1*^*−/−*^ double knockout mouse^99,100^ lacks dystrophin and the myogenic regulator Myod1, which is crucial for proliferation and differentiation of skeletal muscle progenitors during regeneration.^96^ Double *mdx/Myod1*^*−/−*^ knockouts have significantly increased dystrophic disease severity. Animals have markedly reduced muscle mass and myofiber cross sectional area, develop kyphosis by 3–5 months of age and die prematurely at 12 months of age. Consistent with the role of MyoD1 as a regulator of myogenesis, satellite cells from *mdx/Myod1*^*−/−*^ have impaired myogenic capacity. Despite a greater than 2-fold increase compared to *mdx* animals, satellite cells from double knockout animals are unable to contribute to new myofibers due to an increased propensity for self-renewal instead of differentiation. Accordingly, *mdx/Myod1*^*−/−*^ mice have markedly reduced regeneration capacity following injury, with high numbers of mononuclear cells but limited myofiber formation.^100^ Overall, the *mdx/Myod1*^*−/−*^ double knockout mouse model was instrumental in showing the critical role of Myod1 in adult muscle regeneration, as well as the requirement for satellite cells in response to muscle injury

Intriguingly, despite the fact that MYOD1 is not expressed in the heart, *mdx/Myod1*^*−/−*^ mutants developed progressive cardiomyopathy.^99^ Hearts become hypertrophic by 5 months of age, with increases in ventricular diameter as well as regions of cardiomyocyte hypertrophy predominantly in the LV. By 10 months of age fibrosis in hearts becomes apparent, with surviving animals at 12 months displaying extensive fibrotic regions confined primarily to the LV. Functional defects have been noted by echocardiology. These heart phenotypes, despite the lack of MYOD1 involvement in the heart, demonstrate that the systemic changes induced by skeletal muscle myopathy contribute to subsequent cardiomyopathy.

*MYOD1* mutations or alterations in protein expression do not occur in human DMD. In addition, human cases of *MYOD1* mutations are rare, likely due to a requirement for this key transcription factor during development. While *Myod*^*−/−*^ mice are viable, *MYOD1* loss of function mutations in humans are associated with perinatal lethal fetal akinesia, which is characterized by lack of fetal movement, growth retardation and facial defects. The three affected infants studied also had diaphragm and kidney abnormalities, and died shortly after birth due to hypoxic events. Histological analyses were not performed. These early phenotypes underscore the importance of MYOD1 in development, and, in these cases, the inability of MYF5 to compensate for MYOD1 function in humans. In summary, its involvement in human disorders underscores the critical role of MYOD1 in the regulation of the muscle progenitor regenerative response, but not directly in the pathogenesis of DMD.

### Putative cytidine monophosphate-*N*-acetylneuraminic acid hydroxylase-like protein (mdx/Cmah^*−/−*^)

Key cell-surface glycans with variable numbers of carbohydrates mediate extracellular signaling.^101^ Sialic acids are a class of monosaccharides expressed on the terminal ends of many glycan structures. In mammals, these acids take the form of *N*-acetylneuraminic acid (Neu5Ac) and *N*-glycolylneuraminic acid (Neu5Gc).^102^ Glycans on skeletal and cardiac muscle in mice comprise a 50:50 mixture of Neu5Gc and Neu5Ac, whereas humans lack Neu5Gc completely. This difference is caused by a human-specific deletion in the gene putative cytidine monophosphate-*N*-acetylneuraminic acid hydroxylase-like protein (*Cmah)*, which encodes CMP-Neu5Ac hydroxylase, the enzyme that catalyzes the synthesis of Neu5Gc. In order to mimic this deletion, an inactivating deletion was introduced in the mouse *Cmah* gene.

Single *Cmah*^*tm1Avr*^ knockouts (*Cmah*^*−/−*^) have a “humanized” inactivation of CMAH. They exhibit elevated levels of the precursor *N*-acetylneuraminic acid and sialic acid O acetylation, as well as some behavioral, metabolic and wound healing deficits, but no muscle defects.^103^

When crossed with the *mdx* mouse to produce the *mdx/Cmah*^*−/−*^ double knockout, the humanizing *Cmah* mutation leads to a synergistic enhancement of all the classic symptoms of DMD.^101^ Compared to *mdx* controls, *mdx/Cmah*^*−/−*^ mice have significantly increased fibrosis in the quadriceps and gastrocnemius at 6 weeks, the diaphragm at 6 months and, most notably, in the heart at 3 months of age. By 8 months of age, *mdx/Cmah*^*−/−*^ mouse show 70% reduction in speed and 88% reduction in peak force of diaphragm muscle strength compared to *mdx* controls. Importantly, cardiac defects are also observed. Hearts show early necrotic foci by 3 months of age, as well as a 60% reduction in peak force of cardiac trabeculae at 6 Hz compared to WT hearts, though no cardiomyopathy has been noted. Finally, lifespan is significantly reduced, with over half of *mdx/Cmah*^*−/−*^ mice dying by 11 months of age. Although the mice did not manifest the DCM from which DMD patients die, overall, this mouse model set a successful precedent for “humanization” as an approach to more closely approximate the human disease.

### Telomerase RNA component (*mdx*^*4cv*^*/mTR*^*−/−*^)

Although mice have much shorter lifespans than humans (~2 vs. ~75 years), they have much longer telomeres, the protective caps at the ends of chromosomes, that do not shorten appreciably during aging.^104^ Mouse strains vary both in length and number of short telomeres.^105^ The reason for the difference between mice and humans remains unknown.

The length of telomeres dictates replicative lifespan of cells, known as the Hayflick limit. Telomere length is maintained by the enzyme telomerase, which is comprised of the protein telomerase reverse transcriptase and the telomerase RNA component (TERC or *mTR*). Single gene knockout of mTR leads to ubiquitous telomere shortening, which is initiated in the germ line and propagated to subsequent generations at each mating. The Terc^tm1Rdp^ knockout (*mTR*^*−/−*^) manifests a global premature aging phenotype by the fourth generation, *mTR*^*G4*^, including DCM. At second generation, *mTR*^*G2*^, the mTR knockout has no phenotype.^106,107^

**Fig. 2:**
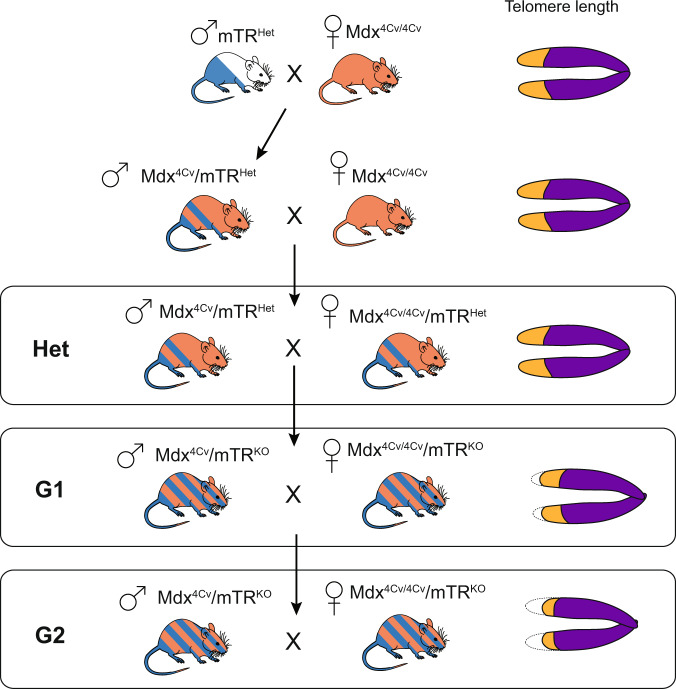
Crossing scheme for generation of the *mdx*^*4cv*^*/mTR*^*G2*^ model of Duchenne Muscular Dystrophy. Breeding begins with an initial cross of a male mTRHet heterozygous animal with a female *mdx*^*4cv/4cv*^ homozygous animal, both of which are available live. The male progeny of this cross are bred with female *mdx*^*4cv/4cv*^ animals. It should be noted that because the dystrophin gene is X-linked, males are inherently hemizygous while females are homozygous. Progeny of the *mdx*^*4cv*^/*mTR*^*Het*^ x *mdx*^*4cv/4cv*^ cross are denoted as “Het”. Male Het animals are heterozygous for the mTR mutation and have the *mdx*^*4cv*^ mutation on the X-chromosome. Given the one functional copy of Terc (mTR), Hets with normal telomere lengths are used as controls. To create the first generation of *mdx4*^*cv*^*/mTR*^*KO*^ (designated as *mdx*^*4cv*^/*mTR*^*G1*^) animals with shortened telomeres, inter-cousin breeding of *mdx*^*4cv*^/*mTR*^*Het*^ is performed to avoid genetic drift.^108,109^ Finally, *mdx*^*4cv*^/*mTR*^*G1*^ animals are crossed, again through inter-cousin breeding, to generate the second generation *mdx*^*4cv*^/*mTR*^*G2*^ animals. *mdx*^*4cv*^/*mTR*^*G2*^ animals have “humanized” telomere lengths that allow the full penetrance of skeletal and cardiac muscle phenotypes

To test if “humanization” of telomere lengths could recapitulate the DMD disease phenotype, the *mdx*^*4cv*^*/mTR*^*G2*^ double knockout mouse was generated (Fig. 2).^108^ By the second generation, when telomeres are somewhat shortened in all tissues, *mdx*^*4cv*^*/mTR*^*G2*^ exhibited a skeletal muscle phenotype resembling DMD including progressive limb and diaphragm muscle wasting, kyphosis, increased fibrosis and calcium deposits, loss of strength. Myoblasts isolated from these mice exhibit shortened telomeres compared to cells in the spleen and testes, as well as compared to their counterparts in *mdx*^*4cv*^*/mTR*^*Het*^ controls.^108^ This telomere shortening led to a defect in muscle regenerative response due to progressive exhaustion and premature depletion of the reservoir of MuSCs necessary to fuel myofiber repair. Importantly, like DMD patients, in *mdx*^*4cv*^*/mTR*^*G2*^ mice progressive DCM is the cause of premature death,^109^ which is not the case for mice in which the *mdx* mutation was bred to different mouse backgrounds.^61^ *mdx*^*4cv*^*/mTR*^*G2*^ mice show decreased cardiac output, progressive fibrosis, and cardiac contractile and conductance dysfunction leading to shortened life span due to heart failure. Specifically, LV enlargement is evident, accompanied by anatomic features of eccentric hypertrophy, including dilation of the ventricular chambers and thinning of the myocardial wall as in late stage DMD patients.^59,110^

The significance of telomere shortening is underscored by the finding that cardiomyocytes of DMD patients have 50% shorter telomeres than cardiomyocytes of unaffected individuals or the cells in the heart that do not require dystrophin for function.^109^ Moreover, the cardiac dysfunction evident by electrocardiogram, ECHO and MRI studies in the *mdx*^*4cv*^*/mTR*^*G2*^ mouse model reflects the reduced systolic function reported for DMD patients using such measures.^111^ Further, telomere shortening, which is known to occur normally during aging in human skeletal myoblasts,^108,112,113^ is also a known feature of DMD muscle cells, which show a diminished proliferative capacity and 14-fold greater shortening in patients compared to healthy controls.^114–116^ These findings together with the synergistic effect of telomere shortening in the *mdx*^*4cv*^*/mTR*^*G2*^ mouse model implicates telomere length in the progression of DMD and suggests that DMD is a disorder of premature cardiovascular and muscle aging. Further, these studies raise the possibility that mice are protected from human diseases such as DMD by the length of their telomeres. Upon comparison with other available mouse models, the *mdx*^*4cv*^*/mTR*^*G2*^ mouse model appears to most closely approximate the human skeletal and cardiac muscle phenotypes of DMD.

## Future directions

With the development of new therapies for DMD on the horizon there is a greater demand to test their efficacy in both muscle and heart. Since the generation of the original *mdx* mouse, which manifests only a mild phenotype, many mouse models have arisen over the years to better recapitulate the disease. Many of these models hinge on additive effects of either structural, or myogenic gene knockouts that are not present in the human disease in order to increase the severity of the phenotype. As a result, the *mdx* model, for all its caveats, is still by far the most widely used.

The years of extensive research in the *mdx* mouse and subsequent double knockout models have vastly increased our understanding of the skeletal muscle component of the disease. The utrophin *mdx/utr*^*−/−*^ knockout demonstrated the compensatory role of utrophin, a developmental homolog to dystrophin that is upregulated in many DMD patients. Also targeting the DGC, the α-dystrobrevin *mdx/dtna*^*−/−*^ double knockout showed the importance of the signaling component of this structural complex, in particular the role of nNOS localization. Focusing on a different sarcolemmal complex, the α7-integrin *mdx/*α7^*−/−*^ double knockout showed the importance of non-DGC elements. Overexpression of α7-integrin decreased the severity of the dystrophy seen in *mdx/Utrn*^*−/−*^ animals, while *mdx/*α7^*−/−*^ animals had more severe skeletal muscle and some cardiac deficits. At the satellite cell level, the *mdx/Myod1*^*−/−*^ knockout demonstrated not only the importance of MyoD1 in adult muscle regeneration, but also the critical role of satellite cells and their progeny in disease progression. Finally, the *mdx/Cmah*^*−/−*^ and *mdx*^*4cv*^*/mTR*^*G2*^ double knockout models established the importance of “humanization” of the *mdx* model. “Humanization” strategies have been shown to act synergistically with other specific disease mutations, such as Werner and ataxia-telangiectasia syndromes^117,118^ in order to better recapitulate a number of human conditions, including cancer, inflammation and infectious disease. The *mdx/Cmah*^*−/−*^ showed that eliminating certain mouse-specific glycan modifications, which occur on many proteins in the DGC and are crucial for signaling, unmasks more severe skeletal muscle and cardiac phenotypes similar to human. Most recently, the *mdx*^*4cv*^*/mTR*^*G2*^ demonstrated that species-specific differences in telomere length account for differences in the regenerative capacity of satellite cells from *mdx* animals vs. humans. In addition, humanization of telomere length uncovered premature telomere shortening in cardiomyocytes as a hitherto unknown characteristic of DMD.

**Fig. 3:**
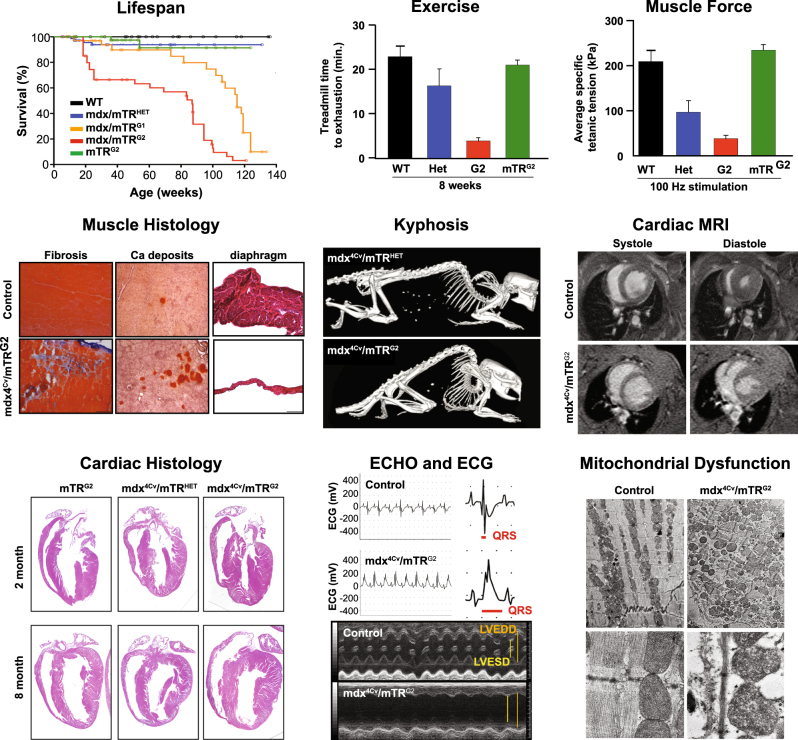
m*dx*^*4cv*^*/mTR*^*G2*^ animal model faithfully recapitulates the skeletal and the cardiac phenotype of DMD. Molecular and functional characterization of skeletal muscle and cardiac phenotypes in the *mdx*^*4cv*^*/mTR*^*G2*^ double knockout mouse. Overall, *mdx*^*4cv*^*/mTR*^*G2*^ exhibited decreased life span. In the skeletal muscle, *mdx*^*4cv*^*/mTR*^*G2*^ exhibited decreased exercise capacity, decreased muscle strength, histological evidence of muscular dystrophy, and kyphosis. In the heart, *mdx*^*4cv*^*/mTR*^*G2*^ displayed cardiac dysfunction as measured by MRI, histology, ECHO, ECG, and electron microscopy. Figure adapted from prior *mdx*^*4cv*^*/mTR*^*G2*^ studies^108,109^

Nevertheless, despite the heroic fundamental insights into DMD disease progression paved by these many mouse models, there are still few tenable therapies. Instead, increased lifespan in DMD patients has been primarily due to advancements in cardiac and respiratory support, which only treat symptoms of the disease. Gene editing strategies, including exon skipping,^71,119–121^ delivery of mini-dystrophin^122^ and direct editing of the genome through CRISPR,^123–125^ show promise in restoring dystrophin expression and skeletal muscle strength in *mdx* animals (see McGreevy 2015 for review).^126^ However, gene-editing constructs must be tailored for the particular mutation of each patient, and, to date, have limited penetrance, especially in the heart. If systematic development of more modular therapies is to progress, the field will require a mouse model more similar to the *mdx* model. Even with improved delivery of promising strategies like gene editing, testing must be done in mice with the full spectrum of DMD pathology. In particular, therapies that address the progressive DCM in DMD necessitate an animal model with both functional, as well as cellular heart defects on par with the human disease. To ensure that our therapies continue to become more effective and sophisticated, so too must our disease models. To date *mdx*^*4cv*^*/mTR*^*G2*^ appears to most closely recapitulate both the skeletal muscle as well as the cardiac DMD phenotypes (Fig. 3), but is cumbersome to generate due to the multiple genotypes that must be maintained. We are therefore pleased to report that the Jackson Laboratories is now providing these mice at the cost of the mdx mouse to researchers worldwide.
