# Author Correction: Humanizing the mdx mouse model of DMD: the long and the short of it

**DOI:** 10.1038/s41536-020-00112-0

**Published:** 2020-12-21

**Authors:** Nora Yucel, Alex C. Chang, John W. Day, Nadia Rosenthal, Helen M. Blau

**Affiliations:** 1 Baxter Laboratory for Stem Cell Biology, Department of Microbiology and Immunology, Institute for Stem Cell Biology and Regenerative Medicine, Stanford University School of Medicine, Stanford, CA 94305 USA; 2 Department of Neurology, Stanford University, Stanford, CA USA; 3 The Jackson Laboratory, Bar Harbor, ME USA

Correction to: *npj Regenerative Medicine* 10.1038/s41536-018-0045-4, published online 16 February 2018

The original version of the published Article contained an error in the initial cross depicted in Fig. 2. The initial cross should be a male *mTR*^*Het*^ heterozygous animal crossed with a female *mdx*^*4cv/4cv*^ homozygous animal.

This has been corrected in the HTML and PDF version of the Article.

